# *In vitro* Effects of Lemongrass Extract on *Candida albicans* Biofilms, Human Cells Viability, and Denture Surface

**DOI:** 10.3389/fcimb.2016.00071

**Published:** 2016-06-28

**Authors:** Petrus L. B. Madeira, Letícia T. Carvalho, Marco A. B. Paschoal, Eduardo M. de Sousa, Eduardo B. Moffa, Marcos A. dos Santos da Silva, Rudys de Jesus Rodolfo Tavarez, Letícia M. Gonçalves

**Affiliations:** ^1^Post-Graduate Program in Dentistry, CEUMA UniversitySão Luis, Brazil; ^2^Post-Graduate Program in Parasite Biology, CEUMA UniversitySão Luis, Brazil

**Keywords:** human cells, *C. albicans*, lemongrass extract, biofilm

## Abstract

The purpose of this study was to investigate whether immersion of a denture surface in lemongrass extract (LGE) has effects on *C. albicans* biofilms, human cell viability and denture surface. Minimal inhibitory concentration (MIC) and minimal fungicidal concentration (MFC) were performed for LGE against C. albicans. For biofilm analysis, discs were fabricated using a denture acrylic resin with surface roughness standardization. *C. albicans* biofilms were developed on saliva-coated discs, and the effects of LGE at MIC, 5XMIC, and 10XMIC were investigated during biofilm formation and after biofilm maturation. Biofilms were investigated for cell counting, metabolic activity, and microscopic analysis. The cytotoxicity of different concentrations of LGE to peripheral blood mononuclear cells (PBMC) was analyzed using MTT. The effects of LGE on acrylic resin were verified by measuring changes in roughness, color and flexural strength after 28 days of immersion. Data were analyzed by ANOVA, followed by a Tukey test at a 5% significance level. The minimal concentration of LGE required to inhibit C. albicans growth was 0.625 mg/mL, while MFC was 2.5 mg/mL. The presence of LGE during biofilm development resulted in a reduction of cell counting (p < 0.05), which made the MIC sufficient to reduce approximately 90% of cells (p < 0.0001). The exposure of LGE after biofilm maturation also had a significant antifungal effect at all concentrations (p < 0.05). When compared to the control group, the exposure of PBMC to LGE at MIC resulted in similar viability (p > 0.05). There were no verified differences in color perception, roughness, or flexural strength after immersion in LGE at MIC compared to the control (p > 0.05). It could be concluded that immersion of the denture surface in LGE was effective in reducing C. albicans biofilms with no deleterious effects on acrylic properties at MIC. MIC was also an effective and safe concentration for use.

## Introduction

*Candida albicans* is the main pathogen responsible for the development of *Candida*-associated denture stomatitis (CADS), an infection commonly observed in denture wearers (Gendreau and Loewy, 2011). If not treated with appropriate hygiene or antifungal treatments, the yeast cells adhere to the acrylic denture surface and are predominantly organized as biofilms (Krom et al., 2014; Goncalves et al., 2015).

Mechanical brushing is a simple and widely used method of hygiene used to control biofilms in dentures (Tan et al., 2014). However, the visual limitations and poor hand function of some denture wearers (Padilha et al., 2007), in combination with the challenges posed by denture design, can limit biofilm removal (Glass et al., 2010). Although chemical immersion solutions have been recommended as complementary methods to mechanical hygiene (Pellizzaro et al., 2012), it has been observed that these treatments also fail to remove *C. albicans* biofilms (Lucena-Ferreira et al., 2013, 2014). In cases where CADS is a persistent infection, applying an antifungal agent becomes a daily routine (e.g., fluconazole and nystatin; Lalla and Dongari-Bagtzoglou, 2014). The drug resistance of *C. albicans* is one of the major problems in combatting the survival and propagation of biofilm and may ultimately lead to treatment failure (Taff et al., 2013; Ramage et al., 2014).

Considering the current challenges with controlling CADS using conventional therapies, the search for alternative antifungal substances has become a trend in the medical literature (Khan and Ahmad, 2012; Taweechaisupapong et al., 2012; Nett, 2014). In the process of developing new pharmacologically active compounds, plants used in folk medicine represent a rational approach to the quest for drugs (Ahmad and Beg, 2001; Duarte et al., 2005; Rios and Recio, 2005; Vieira et al., 2014). Among these plants, *Cymbopogon citratus*, an herb known around the world as lemongrass, has been reported to exert potent inhibitory effects against *Candida* species (Abe et al., 2003; Silva Cde et al., 2008; Irkin and Korukluoglu, 2009; Amornvit et al., 2014; Tadtong et al., 2014). Thus, the application of lemongrass as an effective and easily available antifungal agent could be considered an alternative approach to reducing *C. albicans* biofilms on denture surfaces.

It is important to point out that in most studies, lemongrass susceptibility tests were performed in planktonic cells (Abe et al., 2003; Silva Cde et al., 2008; Irkin and Korukluoglu, 2009; Tyagi and Malik, 2010; Khan and Ahmad, 2012; Amornvit et al., 2014; Boukhatem et al., 2014; Tadtong et al., 2014), while most *C. albicans* cells in the oral cavity were associated with biofilms. It is known that biofilms differ substantially from planktonic cells, particularly in terms of their higher antifungal resistance (Ramage et al., 2014). In addition, investigations about the safety of lemongrass and its possible effects on the mechanical or physical properties of acrylic surfaces (Moffa et al., 2011; Paranhos Hde et al., 2013; de Sousa Porta et al., 2015) are of the utmost importance in deciding whether this herb is a possible auxiliary treatment for CADS. Thus, the purpose of this study was to investigate whether immersing the denture surface in LGE would have an effect on *C. albicans* biofilms, human cell viability and denture surfaces.

## Materials and methods

### Study design

The antifungal activity of LGE against *Candida albicans* planktonic cells was verified by minimal inhibitory concentration (MIC) and minimal fungicidal concentration (MFC). Discs were fabricated using a denture acrylic resin for biofilm analysis. *C. albicans* biofilms were developed on saliva-coated discs in specific culture mediums with LGE at MIC, 5XMIC, or 10XMIC (experimental groups) for 72 h in order to investigate the effects of LGE during biofilm formation. In addition, the effects of LGE on 72 h-mature biofilms were also investigated by immersing them in distilled water (control group) and LGE at MIC, 5XMIC, or 10XMIC (experimental groups) for 8 h. Analyses included cell count, metabolic activity, and microscopic assays. Furthermore, the cytotoxicity effect of different concentrations of LGE on peripheral blood mononuclear cells (PBMC) was analyzed using an MTT assay as well. Substrata measurements were indicated by changes in color, surface roughness, and flexural strength, which were analyzed after 28 days of immersion in LGE. All tests were performed in triplicate on three different days (*N* = 9). Data were analyzed by ANOVA, followed by Tukey's test at 5% of the significance level.

### Vegetal material

#### Collection and botanical identification

The vegetal material of *Cymbopogon citratus* (i.e., lemongrass) was grown in the experimental field of the Federal University of Maranhão, São Luís, Maranhão, Brazil. The sample was collected from November 2013 to August 2014. Exsiccate was prepared and sent to the Ático Seabra Herbarium of the Federal University of Maranhão for botanical identification. Exsiccate was prepared and sent to the Herbarium “Atticus Seabra” of the Federal University of Maranhão to botanical identification (plant voucher #00537).

#### Preparation of the extract

The vegetal material was dried separately in a greenhouse at 37°C air circulation for 48 h then triturated in a cutting mill. The remaining water was evaporated through freeze drying. For 24 h, the dried and crushed lemongrass (200 g) was extracted at room temperature through maceration with ethanol at 70%. That process was repeated four times. The LGE obtained was filtered and then concentrated under reduced pressure in a rotatory evaporator. The dried residue was diluted in sterile saline solution to a final concentration of 200 mg/mL, sterilized through filtration with a 0.22 μg/mL membrane and kept in sterile amber bottles at 4°C for testing purposes.

### *Candida albicans* growth conditions

*Candida albicans* (ATCC 18804) was aerobically cultured from the original broth through incubation in Sabouraud Dextrose Agar (SDA; Difco, Detroit, MI, USA) for 24 h at 35°C. A loop of yeast cells was inoculated into a Yeast Nitrogen Base (YNB; Difco, Detroit, MI, USA) culture medium, supplemented with 100 mM glucose and aerobically incubated under agitation at 35°C. During the exponential growth phase (i.e., after 18–20 h of incubation), the *C. albicans* cells were washed twice with phosphate-buffered saline (PBS, pH 7.2). Inoculum was prepared in an YNB medium and optically standardized at a mean of ~10^7^ cells/mL (*OD* = 0.25 at 520 nm).

### Susceptibility tests

The results of MIC and MFC tests were determined using M27-A3 standards (CLSI, 2008). For all assays, fluconazole (reference powder; Sigma Aldrich Co., St. Louis, MO, USA) was used as a positive control (Goncalves et al., 2012). A negative control and a solvent (ethanol) control were also used.

#### Minimal inhibitory concentration

An MIC assay was performed with a microdilution test using a 96-well-culture plate. The stock solutions of LGE were diluted and transferred into the first well and serial dilutions were performed to obtain concentrations in the range of 100–0.19 mg/mL. The previously prepared *C. albicans* standard suspension (~10^7^ cells/mL) was adjusted in an RPMI 1640 medium so that it contained ~10^3^ cells/mL. It was then added to all wells. The plate was incubated at 37°C for 48 h, and MIC was defined as the lowest concentration of LGE that could inhibit visible growth.

#### Minimal fungicidal concentration

The non-visible growth wells verified in the MIC test were cultured on SDA plates and incubated at 37°C for 48 h. MFC was considered to be the lowest concentration of LGE that could kill fungal cells (≥99% of fungal death).

### Antifungal activity in biofilms

#### Discs fabrication

Round-shaped discs (10-mm diameter, 2-mm thickness) were fabricated using a water-bath polymethylmethacrylate (PMMA) acrylic resin (VipiCril Plus; VipiCril, São Paulo, Brazil) according to manufacturers' instructions. The discs were prepared using a stainless matrix with standardized dimensions. Processed discs were immersed in distilled water for 48 h at 37°C to allow for the release of residual monomers (Moura et al., 2006). To simulate the inner side of a denture, the disc surfaces were ground in a horizontal polisher (model APL-4; Arotec, São Paulo, Brazil) using progressively smoother aluminum oxide papers (320, 400, and 600 grit). The surface roughness of all discs was analyzed using a rugosimeter (Mitutoyo Corp., Tokyo, Japan) with an accuracy close to 0.01 μm and calibrated at cutoff values of 0.8, 2.4 mm percussion of measure and 0.5 mm/s. The mean of three measurements for each disc was calculated, and the surface roughness was standardized at 0.31 ± 0.05 μm. After these measurements, the discs were ultrasonically cleansed in purified water for 20 min and disinfected with 1.0% sodium hypochlorite for 3 min to remove any contaminants or artifacts.

#### Biofilm development

To mimic the oral cavity, the discs were coated with human salivary pellicles prior to biofilm development. Saliva was collected from a healthy volunteer who provided written formal consent according to a protocol approved by the Ethics Committee in Research of Ceuma University (#105/2014). Saliva was collected in an ice-chilled polypropylene tube during masticatory stimulation with a flexible film, clarified by centrifugation (10,000 × g for 5 min at 4°C) with the supernatant filter, sterilized, and used immediately.

Under aseptic conditions, each disc was placed in a 24-well-culture plate. An aliquot of saliva was then added to each well. The plate was aerobically incubated for 1 h at 37°C to form the salivary pellicle. Saliva-coated discs were then transferred to a 24-well-culture plate, and a pre-prepared *C. albicans* standard suspension (~10^7^ cells/mL) was added to each well and aerobically incubated at 37°C for 1.5 h (adhesion phase). The discs were carefully washed with PBS and transferred to a new 24-well-culture plate containing an YNB 100 mM glucose culture medium for 24 h. At the end of this period, the discs were washed with PBS, and a new fresh medium was added and aerobically incubated for 72 h at 37°C.

The effects of LGE in *C. albicans* biofilms were investigated at two distinct times: while the biofilm was forming and after it matured. In the first step, LGE was added daily to the fresh culture medium at MIC, 5XMIC, or 10XMIC for 3 consecutive days (72 h) to allow biofilm to develop. The goal of the second step was to verify the effects of LGE in biofilms matured for 72 h and submit them to the groups cited above for 8 h. The analysis was carried out through biofilm cell counting, metabolic activity, and fluorescence microscopy.

#### Cell counting

For cell counting, biofilm discs were washed twice with PBS and sonicated (7 W, for 30 s) to allow for disruption of the biofilm structure. Then the sonicated solutions were serially diluted in PBS and plated in triplicate onto SDA. Plates were aerobically incubated for 24 h at 37°C, and the yeast cells were counted and converted into cells/mL units with the support of a stereomicroscope.

#### Metabolic activity

The metabolic activity was determined with a modified XTT assay protocol (Moura et al., 2006; da Silva et al., 2008). Accordingly, biofilm discs were placed in a 24-well-culture plate with XTT solution (PBS supplemented with 200 mM glucose, 1 mg/mL XTT, and 0.4 mM menadione), and the plates were protected from light and incubated for 3 h at 37°C. Colorimetric changes in the supernatant were analyzed with the support of a spectrophotometer adjusted to 492 nm.

#### Microscopic analysis

The biofilm's structure was evaluated using fluorescence microscopy (Axio Imager Z2; Carl Zeiss, Oberkochen, Germany). The discs of biofilm were washed with PBS twice, stained with SYTO-9 and propidium iodide with the Live/Dead BacLight viability kit (Invitrogen-Molecular Probes, Eugene, OR, USA), incubated for 20 min at 37°C and protected from light. At least five randomly chosen optical fields were examined for each disc using the 63.4x immersion lens.

### Human cell viability

Peripheral blood mononuclear cells (PBMCs) were collected from healthy human volunteers (non-smoking donors who had not taken any drugs for at least 15 days prior to sampling, aged 18–35 years old) who provided written formal consent according to a protocol approved by the Ethics Committee in Research of Ceuma University (#105/2014). Cells were isolated using the standard method of density-gradient centrifugation over Histopaque®-1119. PBMCs were washed and suspended in a supplemented DMEN culture medium with added fetal bovine serum 10%, 100 μg/ml streptomycin, and 100 U/ml penicillin. PBMCs were plated in 96-well-plates (2 × 10^5^ cells/well in 100 μL) and added to 100 μL of PBS or LGE at MIC, 1/2MIC, 5XMIC, or 10XMIC. These sets were incubated at 37°C in a 5% CO_2_ atmosphere for 24 h. At the end of the culture, an aliquot of 3-(4,5-dimethylthiazol-2-yl)-2,5-diphenyl tetrazolium bromide (MTT, 2 mg/mL in PBS) was added to each well, incubated for another 4 h at 37°C and protected from light. At this point, all of the solutions consisted of complete mediums, and the MTT was discarded. The formazan crystals were dissolved in DMSO (100 μL), and the absorbance at 540 nm was determined using a spectrophotometer.

### Effects of LGE on acrylic resin

Acrylic resin discs were immersed in distilled water (the control), LGE at MIC or LGE at 5XMIC. Discs were incubated at 37°C for 28 days. The immersion medium was changed daily, and the disc was rinsed in sterile distilled water and dried with absorbent paper. The tests were performed after 0, 7, 14, 21, and 28 days of immersion.

#### Color perception

At each testing time, the discs were positioned under a silicon mold with an opening aimed to contact the samples to a spectrophotometer (EasyShade Advanced 4.0; Wilcos, Germany). This mold was used in order to accurately reposition and measure the colors of the disc surface. The color measurements were obtained using the CIE L^*^a^*^b^*^ color system following a previously established protocol (de Sousa Porta et al., 2015). The total color alteration (ΔE) was calculated using on the following equation: ΔE^*^ = [(ΔL^*^)^2^ + (Δa^*^)^2^ + (Δb^*^)^2^]^1∕2^.

#### Surface roughness

At the same tested time, the discs were subjected to a surface roughness measurement (Surftest SJ-201P rugosimeter, Mitutoyo Corp.). For each sample, three readings were performed in the corresponding regions at 2.4 mm in length, a cutoff value of 0.8 mm, and a speed of 0.5 mm/s. The roughness of each disc was calculated using the arithmetic mean of three measurements (μm). The measurement of surface roughness (ΔRa) was obtained based on the difference in roughness before and after immersion.

#### Flexural strength

For the flexural strength assay, rectangular acrylic resin discs (65 × 10 × 3 mm) were fabricated, and a stainless matrix was used to standardize dimensions. The flexural strength (*S*) was measured using a three-point bending test in a DL 2000 universal testing machine (EMIC, São José dos Pinhais, PR, Brazil) at a crosshead speed of 5 mm/min. The discs were subjected to flection until they fractured using a previously established protocol (Paranhos Hde et al., 2013). Flexural strength was calculated using the formula *S* = *3PL/2bd*^2^, where *S* is flexural strength, *P* is the peak load applied, *L* is the span length, *b* is the disc width, and *d* is the disc thickness. The results were expressed in MPa.

### Statistical analysis

The results were statistically analyzed by the SAS/LAB software package (SAS Software, version 9.0; SAS Institute Inc., Cary, NC, USA). Assumptions about the equality of variances and the normal distribution of errors were checked. Data were transformed, as suggested by the software. Data concerning cell counts, metabolic activity, the viability of PBMC cells, and flexural strength were analyzed using a one-way ANOVA followed by Tukey's HSD test, in which the immersion treatment was treated as the study factor. Surface roughness and color alteration data were analyzed by two-way ANOVA for repeated measures followed by Tukey's HSD test, in which the treatment and immersion periods were considered to be study factors. The significance level was set to 5% for all tests.

## Results

The MIC and MFC of LGE observed for planktonic cells were 0.625 mg/mL and 2.5 mg/mL, respectively, while fluconazole was 0.5 and >64 μg/mL.

**Figure 1:**
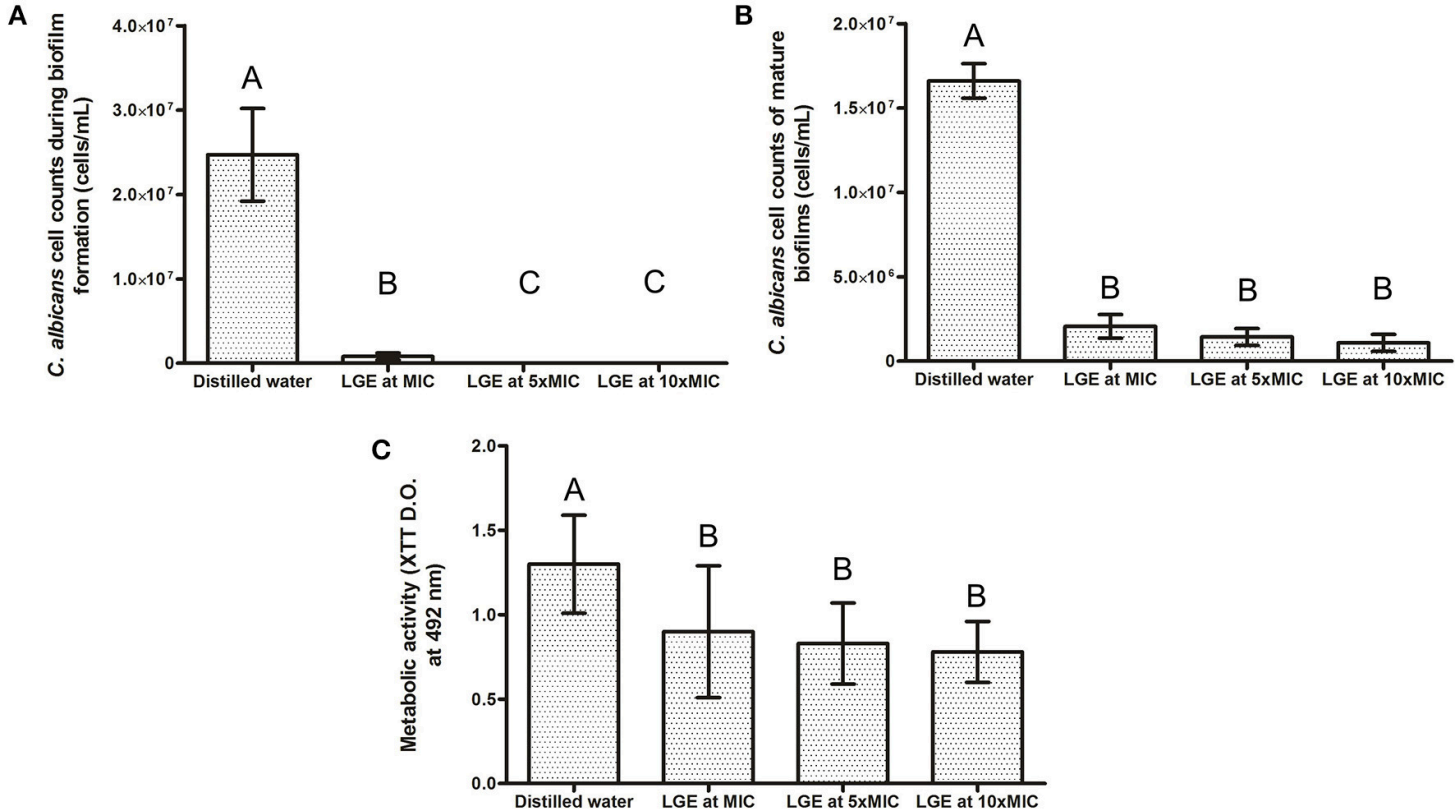
**Effect of LGE on *C. albicans* biofilm. (A)** Number of cell counts when LGE was daily added to the fresh culture medium at different concentrations (MIC, 5XMIC, or 10XMIC) during 72 h biofilms formation; **(B)** Number of cell counts of a 72 h biofilm immersed for 8 h at different concentrations LGE (MIC, 5XMIC, or 10XMIC); **(C)** Metabolic activity of biofilms when mature biofilms were exposed to LGE. Different upper case letters represents statistically significant differences between groups (ANOVA one-way followed by Tukey test, *p* < 0.05).

It was observed that LGE exposure during biofilm development reduced the cell count compared to control group (*p* < 0.05). In addition, LGE at MIC was enough to reduce approximately 90% of biofilm cells (*p* < 0.0001), while LGE at 5 or 10XMIC achieved almost complete eradication (>99%) of *C. albicans* biofilm (Figure 1A). For mature *C. albicans* biofilms, an 8 h exposure of LGE also had a significant effect, with lower cell counts (*p* = 0.001) and metabolic activity in all studied groups compared to the control group (*p* < 0.05), but with no statistical differences between the experimental groups and the studied variables (*p* > 0.05, Figures 1B,C).

**Figure 2:**
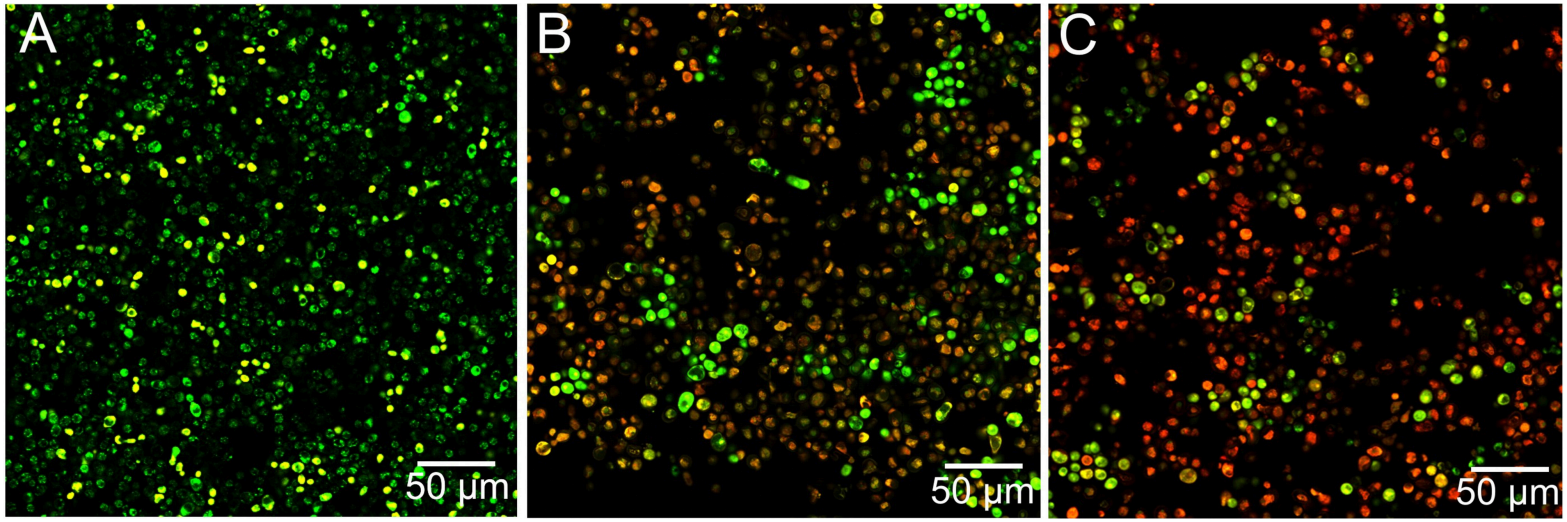
**Representative microscope images of mature *C. albicans* biofilms after 8 h of exposure to LEG**. The combined use of SYTO-9 and propidium iodide effectively labeled both live and dead yeast cells under different experimental conditions: distilled water **(A)**, LEG at MIC **(B)** or LEG at 5XMIC **(C)**. Images showed that mature biofilms exposed to LGE tended to be less densely celled **(B,C)** than the control group **(A)**. Immersion in LGE at MIC **(B)** and 5XMIC **(B)** resulted in a large number of dead cells as well as a large number of black spaces, indicating the dispersion of biofilms.

Representative microscope images of *C. albicans* biofilms were shown in Figure 2. The combined used of SYTO-9 and propidium iodide effectively labeled both live and dead yeast cells under different experimental conditions. Images showed that mature biofilms exposed to LGE tended to be less densely celled than the control group. Immersion in LGE at MIC, 5XMIC, and 10XMIC resulted in a large number of dead cells (stained in red) as well as a large number of black spaces, indicating the dispersion of biofilms.

**Figure 3:**
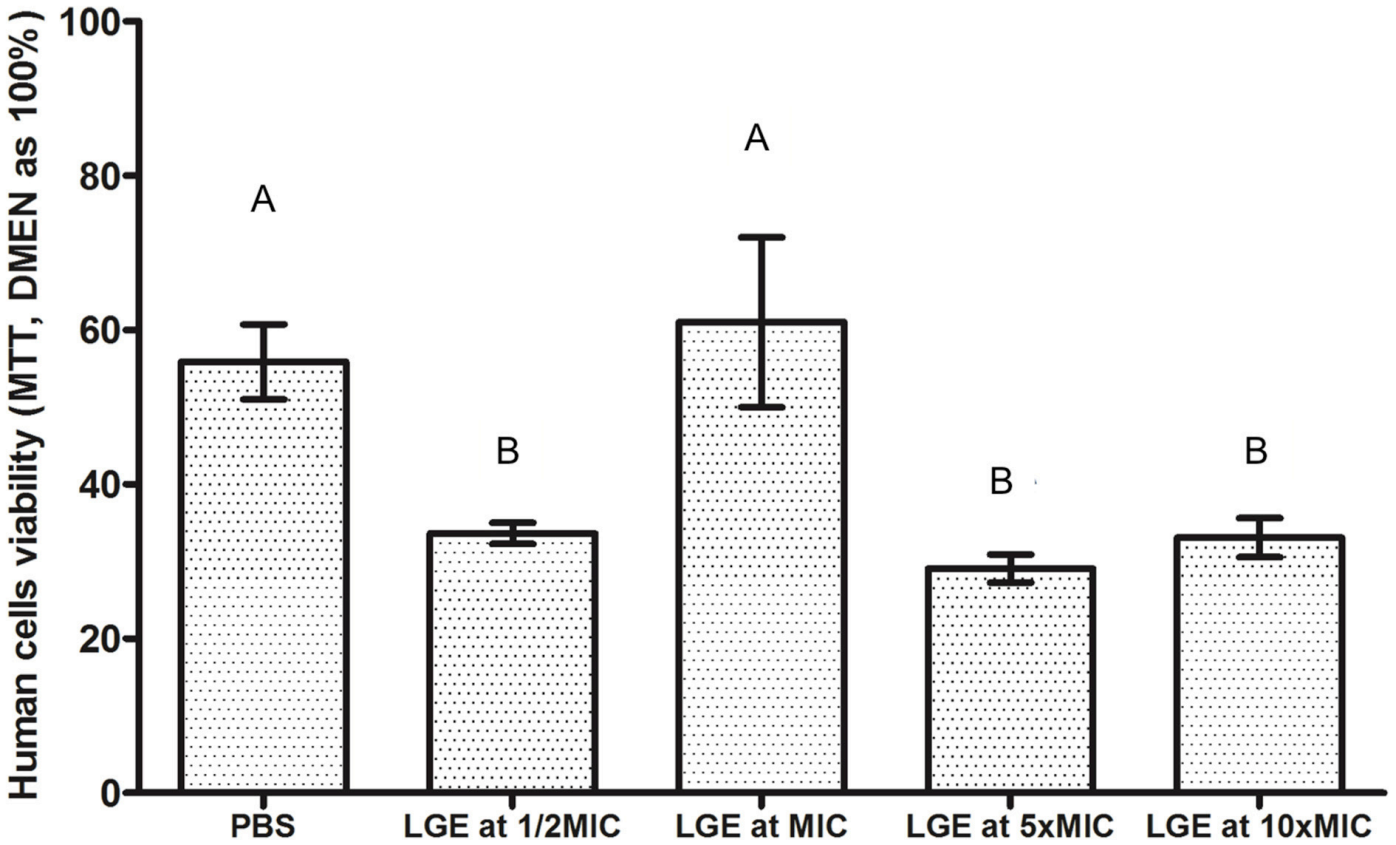
**PBMC cells viability after exposure to different LGE concentrations**. Different upper case letters represents statistically significant differences between groups (ANOVA one-way followed by Tukey test, *p* < 0.05).

If the PBMC culture in DMEN is the standard of 100% viability, it could be observed that LGE significantly reduced its viability (*p* < 0.05). However, when compared to the PBS group, which had no cytotoxic effects, the exposure of human cells to LGE at MIC resulted in similar viability (*p* > 0.05). Exposures to both lower and higher MICs resulted in significant reductions in cell viability, thereby demonstrating a discrete cytotoxicity (*p* < 0.05, Figure 3).

**Figure 4:**
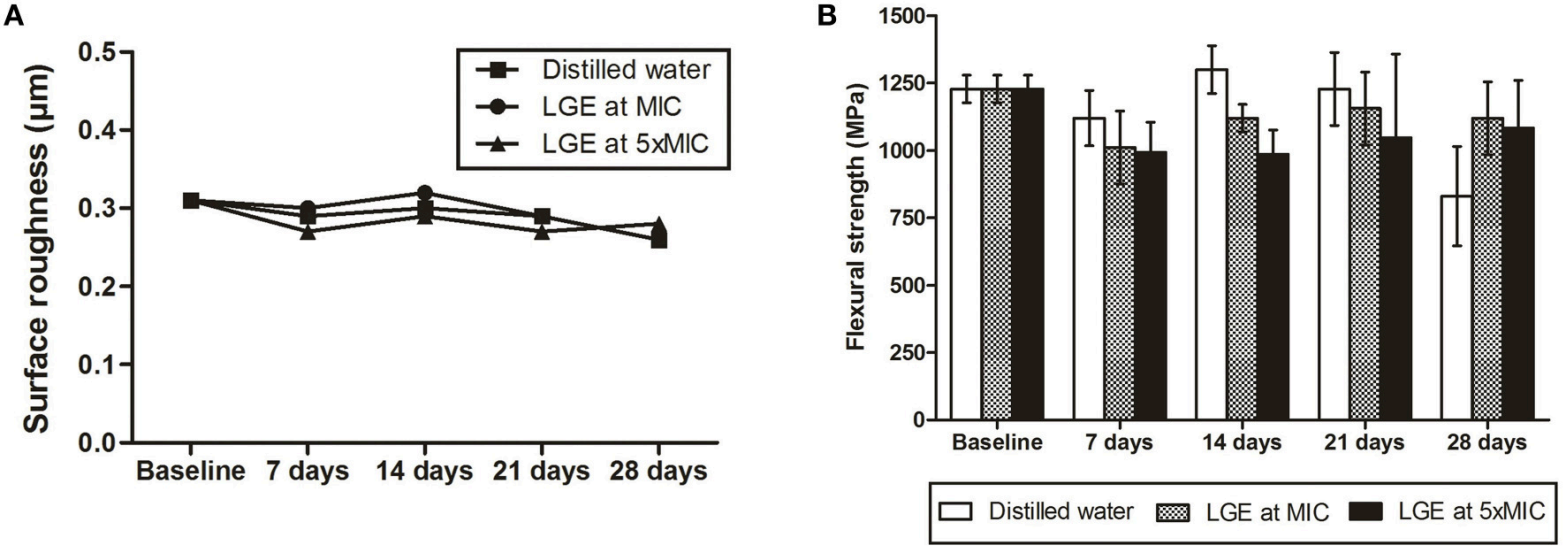
**Analysis of acrylic resin properties after immersion in LGE at different concentrations. (A)** Surface roughness and **(B)** flexural strength after immersion in LGE at different time-points. There were no statistical differences between groups or periods of immersion (ANOVA two-way followed by Tukey test, *p* < 0.05).

Regarding color stability, numerical variations were noticed in the CIEL^*^a^*^b^*^ colorimetric system, but no significant differences between LGE at MIC and the control group were found for ρE values (Table 1, *p* > 0.05). However, after 14 days, a significant color alteration was detected when acrylic resin was immersed in LGE at 5XMIC. The roughness surface values of all evaluated periods of immersion in LGE demonstrated no statistically significant differences between the baseline means (Figure 4A, *p* > 0.05). Also, the mechanical property of flexural strength was not altered after LGE exposure in the different time periods that were tested (Figure 4B, *p* > 0.05).

**Table 1:** **Degree of color difference (ρE) of the acrylic surface after LGE exposure in different periods of time (*n* = 9)**.

	**7 days**	**14 days**	**21 days**	**28 days**
Distilled water	3.24 ± 1.41 (A,a)	3.33 ± 1.82 (A,a)	3.25 ± 1.52 (A,a)	3.22 ± 0.71 (A,a)
LEG at MIC	3.36 ± 0.54 (A,a)	3.05 ± 1.53 (A,a)	3.15 ± 1.86 (A,a)	3.25 ± 1.05 (A,a)
LEG at 5XMIC	3.26 ± 1.38 (A,a)	3.76 ± 0.97 (B,b)	3.88 ± 0.27 (B,b)	4.05 ± 0.61 (B,b)

Different upper case letters indicate statistical significant differences between immersion solutions. Different lower case letters indicate statistical significant differences between immersion periods (ANOVA two-way for repeated measures followed by Tukey test, p < 0.05).

## Discussion

Nowadays, new compounds in all fields of fungal control have stimulated research into plant materials with antifungal properties (Ahmad and Beg, 2001; Duarte et al., 2005; Rios and Recio, 2005; Vieira et al., 2014). *Cymbopogon citratus* (DC) Stapf (*Gramineae*) is an herb known as lemongrass worldwide. The tea made from its leaves is often used in folk medicine as an antispasmodic, analgesic, anti-inflammatory, antipyretic, antimicrobial, diuretic, and sedative (Rios and Recio, 2005). In this study, the extract obtained from lemongrass was examined *in vitro* as an immersion solution to reduce the development of *C. albicans* biofilms on acrylic denture surfaces.

Susceptibility tests suggested that LGE showed fungistatic behavior at 0.625 mg/mL against *C. albicans* planktonic cells and fungicidal activity at 2.5 mg/mL. Regarding MIC results, the following classification for the antimicrobial activity of plant products was proposed: strong inhibitors, MIC up to 0.5 mg/mL; moderate inhibitors, MIC between 0.6 and 1.5 mg/mL; and weak inhibitors, MIC above 1.6 mg/mL (Aligiannis et al., 2001). Regarding this classification, the LGE extract was considered to be a moderate inhibitor, which corroborates the results obtained in this study.

The antifungal effects of lemongrass in several formulations (e.g., citral, vapor phase, essential oil, or hydro-alcoholic extracts) are well-established, and the present data corroborates the literature (Abe et al., 2003; Silva Cde et al., 2008; Irkin and Korukluoglu, 2009; Korenblum et al., 2013; Amornvit et al., 2014; Tadtong et al., 2014). Considering that most *Candida* cells in the oral cavity are associated with biofilms, which differ substantially from planktonic cells due to their higher antifungal resistance (Ramage et al., 2014), investigating the behavior of cells organized in biofilms when they are exposed to LGE is of the utmost importance.

As a starting point, it was observed the effects of LGE during biofilm formation. Thus, LGE was added to a culture medium at different concentrations, and cell quantification was performed after 72 h of development. A more significant reduction was observed in experimental groups at 5XMIC, a concentration sufficient for eliminating biofilm cells. The fact that biofilms were grown in the constant presence of LGE could lead the extract to exert a toxic effect. Several mechanisms have been proposed to explain vegetal antifungal activity, including disruption of the fungi cell structure, thereby causing leakage and cell death; blockage of membrane synthesis; and inhibition of spore germination, fungal proliferation, and cellular respiration (Wilkinson and Cavanagh, 2005). Therefore, investigating the chemical composition of lemongrass allow us to suggest that the presence of aldehydes, which are known for their antibacterial and fungitoxic properties (Wilkinson and Cavanagh, 2005; Khan and Ahmad, 2012; Machado et al., 2012), could explain the outcomes that were achieved.

We investigated the effects of LGE in a 72 h-mature biofilm developed on an acrylic denture surface in order to simulate an overnight denture soaking period of 8 h. Distilled water was employed as a control group, since it is indicated for denture immersion during sleep. These results demonstrated that 8 h of immersion in LGE at MIC were sufficient to reduce the metabolic activity and number of cells compared to the control group. This decrease in a short exposure time could also be explained by the presence of oxygenated monoterpenes aldehydes in the vegetal composition. Citral (3,7-dimethyl-2,6-octadienal) is the major monoterpene present in lemongrass (65–85%), and it is a mixture of two isomeric aldehydes, geranial (*trans*-citral, citral A), and neral (*cis*-citral, citral B), that possess significant antimicrobial activity (Machado et al., 2012), as discussed earlier. Besides this, lemongrass could also contain other antifungal monoterpene hydrocarbons, such as camphene and limonene (Tyagi and Malik, 2010). The fungicidal effects of LGE could be confirmed by the increased number of dead cells observed in microscopic images. Future investigations should take into account the exact molecular mechanisms that explain the death of fungal cells in the presence of LGE.

Maintenance of human cell viability after LGE exposure should be determined to ensure its safe use in future clinical studies. Though the inflammation observed in CADS is mainly characterized by lymphocytes and monocytes (Sinha et al., 2014), assessing the cytotoxic potential of LGE to target these cells is relevant. This study demonstrated that LGE at MIC provided a cellular viability similar to PBS (the control group), although this solution is well-known to be non-toxic to human cells due to its osmotic capacity. Although concentrations higher than MIC did not completely cause cell death, a discrete cytotoxicity could be observed. Thus, according to our results, MIC seems to be a concentration that ensures that satisfactory biofilm control does not result in significant damage to host cells. Nevertheless, cell types present in oral mucosa, such as keratinocytes and fibroblasts, should be considered in further research.

It has been shown that immersion in certain chemical solutions can affect the color, roughness and strength of denture base resins, which are all directly related to longevity and esthetic (Felipucci et al., 2011; Moffa et al., 2011; Paranhos Hde et al., 2013; Savabi et al., 2013; de Sousa Porta et al., 2015). Although 28 days of LGE immersion seems to be a short period of time compared to the lifespan of a denture, we understand that constant exposure to the solution (changed every day) may significantly age the acrylic matrix, thereby creating a challenging situation for the tested discs. Also, based on the similar microbiological and cytotoxic results of LGE 5XMIC and LGE 10XMIC, the higher concentration was excluded from substratum assays.

Color stability after LGE immersion was evaluated using the CIEL^*^a^*^b^*^ colorimetric system, a uniform three-dimensional system that has been widely used to determine chromatic differences by translating their combinations into mathematical data. In this system, color alteration (ρE) is defined as the relative color change between repeated color evaluations. Given that a color difference of < 3.7 is reported to be clinically imperceptible (Moon et al., 2014) the immersion of acrylic resin in LEG at MIC did not cause a visible color alteration in this study. This result seems very interesting because the main disadvantage of conventional chemical cleansers, such as sodium hypochlorite, is their whitening properties when used for prolonged periods (Moffa et al., 2011; Savabi et al., 2013; Moon et al., 2014). Actually, the LGE at 5XMIC was sufficient to stain the substratum surface after 14 days of immersion. The highest concentration of active compounds in this experimental group, which are green in color, may impregnate the acrylic matrix and cause this alteration.

The surface roughness is an important factor of the adherence and entrapment of microorganisms on acrylic denture materials. Therefore, it is crucial that solutions do not alter such properties, since rougher surfaces could increase biofilm formation (Paranhos Hde et al., 2013). Also, it is undesirable for a solution to interfere with the substratum's mechanical properties. If a treatment negatively affects the resins by decreasing their strength, a greater incidence of denture fractures might occur both outside and inside the mouth (Paranhos Hde et al., 2013; de Sousa Porta et al., 2015). In this study, the immersion of acrylic resin in LGE did not lead to significant changes in surface roughness or flexural strength, which means that in experimental conditions, the solution was safe to use.

## Conclusion

The immersion of denture surfaces in lemongrass extract (LGE) was effective in reducing *C. albicans* biofilms without affecting mechanical and physical substratum properties. Furthermore, human cell outcomes showed that the concentration of the tested extract was effective and safe to use. In addition, these results demonstrated the potential to use lemongrass, as valid disinfectant either pre- or post-biofilm formation, as an alternative substance for controlling *C. albicans* biofilms development on denture surfaces.

## Author contributions

contributed to conception or design: PM, LC, MP, ED, EM, MD, RT, and LG. contributed to acquisition, analysis, or interpretation: PM, LC, MP, RT, and LG. drafted the manuscript: PM, MP, ED, MD, and LG. critically revised the manuscript: PM, LC, MP, ED, EM, MD, RT, and LG. gave final approval: PM, LC, MP, ED, EM, MD, RT, and LG. Agrees to be accountable for all aspects of work ensuring integrity and accuracy: PM, LC, MP, ED, EM, MD, RT, and LG.

### Conflict of interest statement

The authors declare that the research was conducted in the absence of any commercial or financial relationships that could be construed as a potential conflict of interest.
